# Whole exome sequencing in 17 consanguineous Iranian pedigrees expands the mutational spectrum of inherited retinal dystrophies

**DOI:** 10.1038/s41598-021-98677-3

**Published:** 2021-09-29

**Authors:** Atta Ur Rehman, Neda Sepahi, Nicola Bedoni, Zeinab Ravesh, Arash Salmaninejad, Francesca Cancellieri, Virginie G. Peter, Mathieu Quinodoz, Majid Mojarrad, Alireza Pasdar, Ali Ghanbari Asad, Saman Ghalamkari, Mehran Piran, Mehrdad Piran, Andrea Superti-Furga, Carlo Rivolta

**Affiliations:** 1 Division of Genetic Medicine, Lausanne University Hospital and University of Lausanne, Lausanne, Switzerland; 2 Noncommunicable Diseases Research Center, Fasa University of Medical Sciences, Fasa, Iran; 3 Department of Genetics and Genome Biology, University of Leicester, Leicester, UK; 4 Department of Medical Genetics and Molecular Medicine, Faculty of Medicine, Mashhad University of Medical Sciences, Mashhad, Iran; 5 Institute of Molecular and Clinical Ophthalmology Basel (IOB), Basel, Switzerland; 6 Department of Ophthalmology, University of Basel, Basel, Switzerland; 7 Institute of Experimental Pathology, Lausanne University Hospital, University of Lausanne, Lausanne, Switzerland; 8 Medical Genetics Research Centre, Faculty of Medicine, Mashhad University of Medical Sciences, Mashhad, Iran; 9 Division of Applied Medicine, Medical School, University of Aberdeen, Aberdeen, UK; 10 Persian Bayangene Research and Training Institute, Shiraz, Iran

**Keywords:** Rare variants, Medical genetics, Consanguinity

## Abstract

Inherited retinal dystrophies (IRDs) constitute one of the most heterogeneous groups of Mendelian human disorders. Using autozygome-guided next-generation sequencing methods in 17 consanguineous pedigrees of Iranian descent with isolated or syndromic IRD, we identified 17 distinct genomic variants in 11 previously-reported disease genes. Consistent with a recessive inheritance pattern, as suggested by pedigrees, variants discovered in our study were exclusively bi-allelic and mostly in a homozygous state (in 15 families out of 17, or 88%). Out of the 17 variants identified, 5 (29%) were never reported before. Interestingly, two mutations (*GUCY2D*:c.564dup, p.Ala189ArgfsTer130 and *TULP1*:c.1199G > A, p.Arg400Gln) were also identified in four separate pedigrees (two pedigrees each). In addition to expanding the mutational spectrum of IRDs, our findings confirm that the traditional practice of endogamy in the Iranian population is a prime cause for the appearance of IRDs.

## Introduction

Inherited retinal dystrophies/degenerations (IRDs) are Mendelian disorders of the eye that affect approximately 1 in 1500 people worldwide and constitute a major cause of inherited blindness^1^. Mainly characterized by the progressive death of photoreceptor cells in the retina, IRDs present a high degree of genetic and phenotypic heterogeneity^2^. So far, mutations in over 270 genes have been associated with various forms of IRDs (RetNet: https://sph.uth.edu/retnet/; accessed April 17, 2020); however, this list is constantly increasing. Sequencing of all exons and exon–intron boundaries of these genes has successfully contributed to the understanding of the genetic etiology of 50–70% of patients^3–5^. Since a larger proportion of IRDs are caused by recessive mutations^6^, next-generation sequencing (NGS) coupled with homozygosity mapping has further accelerated detection of candidate variants in consanguineous pedigrees^3^.

Close-kin marital unions have long been practiced in the Iranian population as a cultural feature^7^. In spite of a significant decline in consanguinity in Iran in recent years, prevalence of recessive genetic disorders is still high in the country, possibly due to the presence of marked population stratification that favors intra-community marriages^7,8^. As a result, increased genomic homozygosity in specific societies or ethnic groups leads to the clinical observation of the effect of rare founder mutations^7,9^. The present study was performed with the aim of characterizing genetically a cohort of 17 consanguineous Iranian families with IRDs.

## Methods

### Enrollment of families and DNA extraction

This study was approved by the Ethics Committees of all our respective Institutions (the Ethikkommission Nordewest- and Zentralschweiz, the Ethics Committee of Mashhad University of Medical Sciences, the Ethics Commission of the Noncommunicable Diseases Research Center of Fasa University of Medical Sciences, and the Ethics Commission of the Canton de Vaud) and adhered to the principles of the Declaration of Helsinki. All individuals participating in this study were Iranian residents, who agreed in contributing to this study by signing a written informed consent form. Patients were clinically evaluated by local ophthalmologists and their medical records were maintained at their respective hospitals. Approximately 5.0 ml peripheral blood was collected using a EDTA K2 golden vac disposable vacuum blood collection tube (Zhejiang Gondong Medical Technology, China) or were mixed with EDTA anticoagulant (Merck KGaA, Darmstadt, Germany) after sample collection. DNA was extracted from peripheral blood leukocytes following standard protocols. Quantitative assessment of DNA was made using a NanoDrop 1000 Spectrophotometer (Thermo Fisher Scientific, USA), whereas integrity was evaluated by running the DNA samples on a 1% agarose gel. Pedigrees were drawn with the help of HaploPainter^10^.

### Genetic analyses

Exome capture and library preparation was performed on one affected individual per family using the SureSelect Human All Exon v6 kit (Agilent, Santa Clara, CA, USA) and the HiSeq Rapid PE Cluster Kit v2 (Illumina, San Diego, CA, USA), from 2 μg genomic DNA. Whole-exome sequencing (WES) was performed at the Institute of Genomics of the University of Tartu (Estonia) using an Illumina HiSeq (HSQ-700358) instrument. Bioinformatic analyses were performed as described previously^11^. Briefly, raw reads were mapped to the human reference genome (hg19/GRCh37) using the Novoalign software (V3.08.00, Novocraft Technologies). Next, Picard (version 2.14.0-SNAPSHOT) was used to remove duplicate reads and Genome Analysis Toolkit (GATK) (version 3.8) was used to perform base quality score recalibration on both single-nucleotide variants and insertion–deletions. A VCF file with the variants was generated by HaplotypeCaller. They were annotated according to a specific in-house pipeline using mainly ANNOVAR software^12^ (Oct 2019 release) and splicing predictors: spliceAI^13^, MaxEntScan^14^, and Ada and RF scores from dbscSNV^15^. Then, DNA variants were filtered to have less than 1% allelic frequency in ExAC^16^, gnomAD^17^, 1000 Genomes^18^, and GME (GME Variome http://igm.ucsd.edu/gme/index.php). Variants were then retained according to their predicted impact on protein sequence and splicing of preRNA, according to ANNOVAR RefSeq annotation (missense, stopgain, frameshift or non-frameshift indels, or canonical splicing) and splicing predictors (MaxEntScan [minimal change of 6], spliceAI [minimal score of 0.2] and dbscSNV-ADA [minimal score of 0.2]).

Autozygosity mapping was performed on WES data with AutoMap^19^. Nomenclature of all the variants was confirmed through VariantValidator^20^. Clinical significance of the variants was evaluated with the help of publicly available databases such as ClinVar^21^, the Human Gene Mutation Database (HGMD)^22^ and Varsome^23^, as well as according to their frequency in available databases, e.g. the Genome Aggregation Database (gnomAD)^17^. Seven online *in-silico* methods were used to predict the pathogenicity of all variants. The online *in-silico* tools used included MutationTaster^24^, Mutation Assessor^24^, Polymorphism Phenotyping v2 (PolyPhen-2)^25^, Likelihood Ratio Test (LRT)^26^, Sorting Intolerant from Tolerant (SIFT)^27^, PROVEAN^28^, and Combined Annotation Dependent Depletion (CADD)^29^. Furthermore, all candidate variants were compared with data from Iranome, a database containing information from 800 exomes from individuals belonging to eight major ethnic groups in Iran (http://www.iranome.ir/; accessed on April 19, 2020). Finally, Sanger sequencing was performed to validate all potentially pathogenic variants and to establish their causality through strict genotype–phenotype co-segregation within the available family members.

## Results

**Figure 1:**
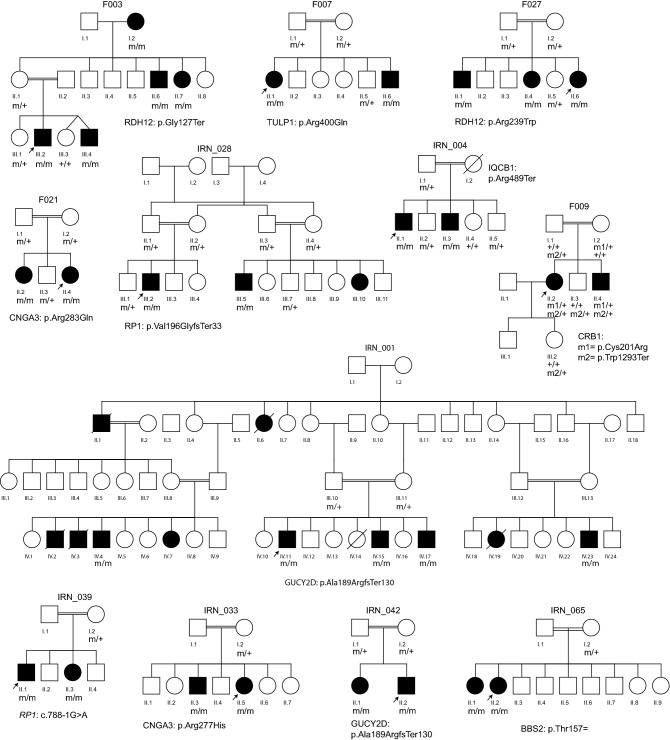

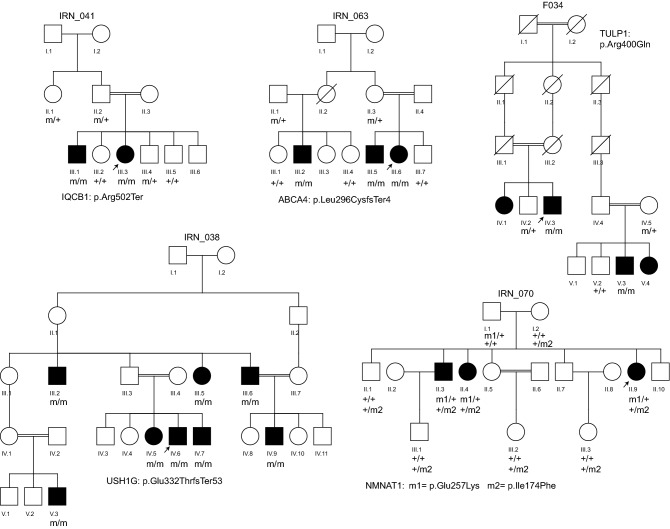
Pedigrees showing genotype–phenotype co-segregation for all detected variants. m, variant identified; + , wild-type allele.

Following WES analysis in 17 probands of Iranian descent, 16 of which were the direct offspring of consanguineous unions (Fig. 1), we identified 17 distinct genetic variants in 11 genes linked to inherited retinal diseases (Tables 1 and 2). Of the 17 pedigrees, two families each were linked to disease-causing variants in *CNGA3*, *GUCY2D*, *IQCB1*, *RDH12*, *RP1*, and *TULP1* genes, while only one family was found with causative variants in either *USH1G*, *ABCA4*, *NMNAT1*, *CRB1*, or *BBS2* genes. The mutational spectrum across these 11 genes comprised 7 missense variants, 4 nonsense variants, 4 small insertion-deletions (Indels) or duplications leading to frameshifts, one canonical splice site variant and one synonymous variant with effect on splicing. As expected, most of the variants in our study were found in a homozygous state (in 15 families out of 17, or 88%). Compound heterozygosity was detected in two families (F009 and IRN_070, Tables 1, 2). Except for one homozygous allele in the *RP1* gene (NM_006269.1:c.788-1G > A) in family IRN_039, all other homozygous pathogenic variants were found in genes that were located inside a so-called run of homozygosity (ROH), generally spanning more than one megabase (Mb) in size.

**Table 1:** List of genetic variants identified in 17 Iranian families.

Family ID	Gene name	Transcript ID	cDNA change	Protein change	Variant type	Zygosity	ROH (Mb)	Autozygome (Mb)
F003	*RDH12*	NM_152443.2	c.379G > T	p.(Gly127Ter)	Nonsense	Hom	17.49	324.54
F007	*TULP1*	NM_003322.5	c.1199G > A	p.(Arg400Gln)	Missense	Hom	21.58	391.04
F009	*CRB1*	NM_201253.2	c.601T > C	p.(Cys201Arg)	Missense	Het	NA	197.05
			c.3879G > A	p.(Trp1293Ter)	Nonsense	Het	NA	197.05
F021	*CNGA3*	NM_001298.2	c.848G > A	p.(Arg283Gln)	Missense	Hom	16.5	312.59
F027	*RDH12*	NM_152443.2	c.715C > T	p.(Arg239Trp)	Missense	Hom	20.78	359.39
F034	*TULP1*	NM_003322.3	c.1199G > A	p.(Arg400Gln)	Missense	Hom	46.06	220.69
IRN_001	*GUCY2D*	NM_000180.3	c.564dup	p.(Ala189ArgfsTer130)	Frameshift	Hom	21.19	425.35
IRN_004	*IQCB1*	NM_001023570.3	c.1465C > T	p.(Arg489Ter)	Nonsense	Hom	24.48	285.78
IRN_028	*RP1*	NM_006269.1	c.584dup	p.(Val196GlyfsTer33)	Frameshift	Hom	4.25	338.69
IRN_033	*CNGA3*	NM_001298.2	c.830G > A	p.(Arg277His)	Missense	Hom	16.5	242.18
IRN_038	*USH1G*	NM_173477.4	c.980_993dup	p.(Glu332ThrfsTer53)	Frameshift	Hom	10.54	228.74
IRN_039	*RP1*	NM_006269.1	c.788-1G > A	r.spl	Splicing	Hom	NA	213.79
IRN_041	*IQCB1*	NM_001319107.1	c.1504C > T	p.(Arg502Ter)	Nonsense	Hom	31.84	165.28
IRN_042	*GUCY2D*	NM_000180.3	c.564dup	p.(Ala189ArgfsTer130)	Frameshift	Hom	2.96	322.99
IRN_063	*ABCA4*	NM_000350.2	c.885del	p.(Leu296CysfsTer4)	Frameshift	Hom	9.94	353.75
IRN_065	*BBS2*	NM_031885.3	c.471G > A	p.(Thr157 =)	Synonymous/splicing	Hom	31.05	185.25
IRN_070	*NMNAT1*	NM_022787.3	c.520A > T	p.(Ile174Phe)	Missense	Het	NA	71.41
			c.769G > A	p.(Glu257Lys)	Missense	Het	NA	71.41

*Hom* homozygous, *Het* heterozygous, *ROH* runs of homozygosity, *NA* not available, *Mb* megabases.

**Table 2:** ClinVar classification, ACMG classification, and other features of all variants identified.

Family ID	Gene name	Transcript ID	cDNA change	Protein change	Previous reports	ClinVar	ACMG	ACMG criteria
F003	*RDH12*	NM_152443.2	c.379G > T	p.(Gly127Ter)	^36^	Pathogenic	Pathogenic	PVS1, PM2, PP1, PP3, PP5
F007	*TULP1*	NM_003322.5	c.1199G > A	p.(Arg400Gln)	^33^	Pathogenic	Likely pathogenic	PM2, PM5, PP1, PP3, PP5
F009	*CRB1*	NM_201253.2	c.601T > C	p.(Cys201Arg)	^32^	Uncertain significance	Likely pathogenic	PM2, PM3, PP1, PP3
			c.3879G > A	p.(Trp1293Ter)	^37^	Pathogenic	Pathogenic	PVS1, PM2, PP1, PP3, PP5
F021	*CNGA3*	NM_001298.2	c.848G > A	p.(Arg283Gln)	^31^	Pathogenic	Likely pathogenic	PM1, PM2, PM5, PP1, PP2, PP3, PP5
F027	*RDH12*	NM_152443.2	c.715C > T	p.(Arg239Trp)	^34^	Likely pathogenic	Likely pathogenic	PM2, PM5, PP1, PP3, PP5
F034	*TULP1*	NM_003322.3	c.1199G > A	p.(Arg400Gln)	^33^	Pathogenic	Likely pathogenic	PM2, PM5, PP1, PP3, PP5
IRN_001	*GUCY2D*	NM_000180.3	c.564dup	p.(Ala189ArgfsTer130)	Novel	NA	Pathogenic	PVS1, PM2, PP1, PP3
IRN_004	*IQCB1*	NM_001023570.3	c.1465C > T	p.(Arg489Ter)	^38^	Pathogenic	Pathogenic	PVS1, PM2, PP1, PP3, PP5
IRN_028	*RP1*	NM_006269.1	c.584dup	p.(Val196GlyfsTer33)	Novel	NA	Pathogenic	PVS1, PM2, PP1, PP3
IRN_033	*CNGA3*	NM_001298.2	c.830G > A	p.(Arg277His)	^35^	Pathogenic	Likely pathogenic	PM1, PM2, PM5, PP1, PP3, PP5
IRN_038	*USH1G*	NM_173477.4	c.980_993dup	p.(Glu332ThrfsTer53)	Novel	NA	Pathogenic	PVS1, PM2, PP1, PP3
IRN_039	*RP1*	NM_006269.1	c.788-1G > A	r.spl	Novel	NA	Pathogenic	PVS1, PM2, PP1, PP3
IRN_041	*IQCB1*	NM_001319107.1	c.1504C > T	p.(Arg502Ter)	^38^	Pathogenic	Pathogenic	PVS1, PM2, PP1, PP3, PP5
IRN_042	*GUCY2D*	NM_000180.3	c.564dup	p.(Ala189ArgfsTer130)	Novel	NA	Pathogenic	PVS1, PM2, PP1, PP3
IRN_063	*ABCA4*	NM_000350.2	c.885del	p.(Leu296CysfsTer4)	^39^	Pathogenic	Pathogenic	PVS1, PM2, PP1, PP3, PP5
IRN_065	*BBS2*	NM_031885.3	c.471G > A	p.(Thr157 =)	^40^	Uncertain significance	Uncertain significance	PM2, PP1, PP3
IRN_070	*NMNAT1*	NM_022787.3	c.520A > T	p.(Ile174Phe)	Novel	NA	Likely pathogenic	PM1, PM2, PM3, PP1, PP2, PP3
			c.769G > A	p.(Glu257Lys)	^30^	Pathogenic	Likely pathogenic	PS3, PM2, PP1, PP2, BP4, PP5

*NA* not available.

Overall, 29% (5 out of 17 different alleles) of the variants reported here were previously unpublished. Similarly, 8 of the total 17 distinct alleles had no gnomAD entry, while the others were all very rare, with no occurrence of homozygous individuals in the gnomAD database (Table 3). Interestingly, we identified two variants, each segregating homozygously in two separate pedigrees: *GUCY2D* (NM_000180.3:c.564dup, p.Ala189ArgfsTer130; families IRN_001 and IRN_042) and *TULP1* (NM_003322.3:c.1199G > A, p.Arg400Gln; families F007 and F034).

**Table 3:** Allele frequencies and pathogenicity scores for all variants identified.

Gene name	Transcript ID	cDNA change	Protein change	gnomAD AF	Iranome AF	SIFT	Polyphen2	LRT	Mutation taster	Mutation assessor	PROVEAN	CADD	MaxEntScan	SpliceAI	dbscSNV ADA	dbscSNV RF
*ABCA4*	NM_000350.2	c.885del	p.(Leu296CysfsTer4)	2.0 × 10^–5^	NA	NA	NA	NA	NA	NA	NA	24.1	NA	NA	NA	NA
*BBS2*	NM_031885.3	c.471G > A	p.(Thr157 =)	1.4 × 10^–5^	NA	NA	NA	NA	NA	NA	NA	NA	Disruption of donor site from 8.54 to 2.27	Disruption of donor site (0.8794)	1	0.96
*CNGA3*	NM_001298.2	c.848G > A	p.(Arg283Gln)	6.4 × 10^–5^	NA	D	D	D	A	H	D	28.8	NA	NA	NA	NA
*CNGA3*	NM_001298.2	c.830G > A	p.(Arg277His)	2.4 × 10^–5^	NA	D	D	D	D	H	D	28.6	NA	NA	NA	NA
*CRB1*	NM_201253.2	c.601T > C	p.(Cys201Arg)	1.2 × 10^–5^	NA	D	D	NA	D	H	D	23.3	NA	NA	NA	NA
*CRB1*	NM_201253.2	c.3879G > A	p.(Trp1293Ter)	NA	NA	NA	NA	NA	D	NA	NA	54	NA	Disruption of acceptor site (0.5395)	0.9997	0.91
*GUCY2D*	NM_000180.3	c.564dup	p.(Ala189ArgfsTer130)	NA	NA	NA	NA	NA	NA	NA	NA	25.7	NA	NA	NA	NA
*IQCB1*	NM_001023570.3	c.1465C > T	p.(Arg489Ter)	2.8 × 10^–5^	NA	NA	NA	N	A	NA	NA	36	NA	NA	NA	NA
*IQCB1*	NM_001319107.1	c.1504C > T	p.(Arg502Ter)	8.0 × 10^–6^	NA	NA	NA	D	A	NA	NA	36	NA	NA	NA	NA
*NMNAT1*	NM_022787.3	c.520A > T	p.(Ile174Phe)	NA	NA	D	D	D	D	H	D	22.9	NA	NA	NA	NA
*NMNAT1*	NM_022787.3	c.769G > A	p.(Glu257Lys)	6.9 × 10^–4^	1.2 × 10^–3^	T	B	D	D	L	N	22.9	NA	NA	NA	NA
*RDH12*	NM_152443.2	c.379G > T	p.(Gly127Ter)	NA	NA	NA	NA	D	A	NA	NA	39	NA	NA	NA	NA
*RDH12*	NM_152443.2	c.715C > T	p.(Arg239Trp)	NA	NA	D	D	D	D	H	D	24.9	NA	NA	NA	NA
*RP1*	NM_006269.1	c.584dup	p.(Val196GlyfsTer33)	NA	NA	NA	NA	NA	NA	NA	NA	32.0	NA	NA	NA	NA
*RP1*	NM_006269.1	c.788-1G > A	r.spl	NA	NA	NA	NA	NA	D	NA	NA	29.8	Disruption of donor site from 1.95 to -6.80		0.9999	0.854
*TULP1*	NM_003322.3	c.1199G > A	p.(Arg400Gln)	7.1 × 10^–6^	NA	D	D	D	D	M	D	29.9	NA	NA	NA	NA
*USH1G*	NM_173477.4	c.980_993dup	p.(Glu332ThrfsTer53)	NA	NA	NA	NA	NA	NA	NA	NA	26.7	NA	NA	NA	NA

*AF* allele frequency, *D* deleterious (SIFT, LRT, PROVEAN) or disease_causing (mutation taster) or damaging (Polyphen 2), *B* benign, *T* tolerated, *N* neutral, *A* disease_causing_automatic, *H* high, *L* low, *M* medium, *NA* not available.

All 7 missense variants with their predicted pathogenicity scores are listed in Table 3. While 6 of them were previously known to be pathogenic, including *NMNAT1* (p.Glu257Lys)^30^, *CNGA3* (p.Arg283Gln)^31^, *CRB1* (p.Cys201Arg)^32^, *TULP1* (p.Arg400Gln)^33^, *RDH12* (p.Arg239Trp)^34^, and *CNGA3* (p.Arg277His)^35^, the remaining one, *NMNAT1* (p.Ile174Phe), was novel. Additionally, segregation analysis within the available family members using Sanger sequencing revealed strict genotype–phenotype correlation for all nonsynonymous variants.

Furthermore, we identified four already published nonsense pathogenic variants in our cohort, including *RDH12* (p.Gly127Ter)^36^, *CRB1* (p.Trp1293Ter)^37^, *IQCB1* (p.Arg489Ter)^38^, and *IQCB1* (p.Arg502Ter)^38^. Similarly, we identified three novel frameshift variants, in addition to the previously known frameshift in the *ABCA4* gene (p.Leu296CysfsTer4)^39^. Novel frameshift variants comprised *GUCY2D* (p.Ala189ArgfsTer130), *RP1* (p.Val196GlyfsTer33), and *USH1G* (p.Glu332ThrfsTer53).

Lastly, we detected a novel canonical splice site variant in the *RP1* gene (NM_006269.1:c.788-1G > A) in family IRN_039, and a known pathogenic synonymous change predicted to alter splicing in the *BBS2* gene (NM_031885.3:c.471G > A;p.Thr157 =) in family IRN_065^40^.

## Discussion

Consanguinity is a major risk factor for the occurrence of rare recessive Mendelian disorders, yet it is a long-lived social practice in many Asian and African countries. In Iran, the second most populated country in the Middle East, 37.4% of all marriages are between consanguineous partners. Of these, 19.3% occur between first cousins and 18.1% involve second cousins^7^.

In this work, we used consanguinity as a means to facilitate identification of mutations in IRD cases, through an autozygome-guided NGS approach. Consistent with the high level of consanguinity displayed by the Iranian population, we observed a recessive inheritance pattern in all our cases, with the largest majority of them carrying indeed homozygous pathogenic variants in known IRD genes. With only one exception, all genes carrying homozygous pathogenic variants resided inside runs of homozygosity, thus supporting earlier studies that highlighted the importance of homozygosity mapping in consanguineous families^41–46^. Nevertheless, compound heterozygous patients were also identified, with mutations in *CRB1* and *NMNAT1*. Interestingly, these patients (from families F009, and IRN_070, respectively) also had relatively lower values of overall genomic homogeneity (197, and 71 Mb, respectively, over an average of 280.0 Mb in the cohort as a whole). The appearance of compound heterozygosity in the Iranian population is not unprecedented, and an earlier study suggested that *CRB1* is a commonly mutated gene in Iranian patients with non-syndromic IRDs^43,47^. Interestingly, our cohort did not include any instance of variants in *USH2A*, although mutations in this gene are considered to be among the most frequent causes of Usher syndrome or non-syndromic retinitis pigmentosa (RP)^48^.

The mutational spectrum in our cohort comprised 1 synonymous (with predicted effect on splicing), 1 splice change, 7 missense, 4 nonsense, and 4 frameshift variants. To establish pathogenicity of the novel missense variants we heavily relied on data from existing literature and the ACMG guidelines. Lastly, we assessed the status of each variant by comparing them with the Iranome database, to filter out common variants specific to Iranian population.

Unlike missense substitutions, the majority of nonsense and frameshift DNA changes can be considered as *bona fide* deleterious mutations, since they mostly constitute loss-of-function (pLoF) alleles in genes where this pathogenicity mechanism is well known (criteria PVS1 of ACMG guidelines). We therefore classified all of them as such, based on this feature and the fact that they were all either absent or present at an extremely low frequency in the gnomAD database.

We also found a synonymous change in the *BBS2* gene (c.471G > A, p.Thr157 =) co-segregating with Bardet-Biedl syndrome in one family (IRN_065) and reported in three previous studies^40,49,50^. Due to the high nucleotide conservation and its localization at an exon–intron boundary, it is possible that the c.471G > A substitution may impair the correct splicing of *BBS2* pre-mRNA. Indeed, all splicing predictors tested (AdaBoost and RandomForest from dbscSNC, MaxEntScan, and spliceAI) indicated a high impact on splicing and disruption of the 5’ site. Our findings thus provide additional support to the potential pathogenicity of this apparently neutral variant. It is worthwhile to mention here that the majority of the previously reported patients with the p.Thr157 = mutation originated from Middle Eastern countries, such as Lebanon and Iran^49,50^. Although this variant perfectly co-segregates with disease in family IRN_065 and has been described in previous reports in association with Bardet-Biedl syndrome^40,49,50^, there is still a chance that it could represent a benign DNA change, detected in homozygosity in our patients by virtue of their ethnical origin. Additional functional studies are needed to definitely confirm its pathogenic role in syndromic IRD.

Since geographic isolation and consanguinity-driven genomic homozygosity lead to the enrichment of rare founder mutations in specific societies or ethnic groups^7,9,51,52^, the presence of such mutations in our cohort of patients from related families is not surprising. Similar to other reports^53–55^, we identified two mutations that were present in more than one pedigree. The first, p.Ala189ArgfsTer130 in *GUCY2D*, was shared by two families originating from the Fars province in the Southwest of Iran and was found in a common ROH of 3.0 Mb with an identical haplotype. The second was a homozygous missense variant (p.Arg400Gln) in the *TULP1* gene, detected in two families from the Razavi Khorasan province, in Northeastern Iran, again in a common ROH of 21.5 Mb with an identical haplotype. This latter variant has been previously reported in an Indian family in a homozygous state^33^.

In summary, this work extends current knowledge about the genetic landscape of IRDs in Iran and, in line with previous studies, supports the evidence that homozygosity mapping is an effective tool for uncovering rare genomic variants in consanguineous pedigrees with rare recessive disorders. Most importantly, we hope that our data would contribute to better molecular diagnosis and access to future gene therapy trials in Iran.
